# No role for glutathione S-transferase genotypes in Caucasian esophageal squamous cell or adenocarcinoma etiology: an European case–control study

**DOI:** 10.1186/1471-230X-13-97

**Published:** 2013-06-03

**Authors:** Polat Dura, Jody Salomon, Rene HM Te Morsche, Hennie MJ Roelofs, Jon O Kristinsson, Theo Wobbes, Ben JM Witteman, Adriaan CITL Tan, Joost PH Drenth, Wilbert HM Peters

**Affiliations:** 1 Department of Gastroenterology, Radboud University Nijmegen Medical Center, P.O. Box 9101, 6500 HB, Nijmegen, The Netherlands; 2 Department of Surgery, Radboud University Nijmegen Medical Center, Nijmegen, The Netherlands; 3 Department of Gastroenterology, Hospital Gelderse Vallei, Willy Brandtlaan 10, 6717, RP Ede, The Netherlands; 4 Deparment of Gastroenterology, Canisius-Wilhelmina Hospital, Weg door Jonkerbos 100, 6532 SZ, Nijmegen, The Netherlands

## Abstract

**Background:**

Identifying and monitoring high-risk patients can aid the prevention of esophageal cancer (EC). The interaction of environmental risk factor exposure and genetic susceptibility may contribute to the etiology of EC. Biotransformation enzymes such as Glutathione S-Transferases (GSTs ) detoxify mutagenic and genotoxic compounds and therefore control the rate of detoxification of carcinogens. Functional polymorphisms in the genes coding for GSTs alter their enzyme activity *in vitro*, and were reported to modify EC risk in Asians. We hypothesized that altered enzyme activity *GST* genotypes influence the susceptibility for esophageal adeno- (EAC) and squamous cell carcinoma (ESCC) in Caucasians.

**Methods:**

We performed a case–control study including 440 Caucasian patients with EC and 592 healthy Caucasian controls matched for age and sex. Functional polymorphisms were selected and genotypes were determined in GST classes Alpha, Mu, Theta and Pi by means of polymerase chain reaction. Genotypes were classified into predicted high, intermediate and low enzyme activity categories based on *in vitro* activity data. The distribution of the activity genotypes were compared between patients with EAC or ESCC, and controls. Odds ratios (OR) with 95% confidence intervals (CI) were calculated by logistic regression analyses. Gene-gene interactions were tested and for comparison purposes, the predicted low and intermediate activity genotypes were combined. Genotypes with similar risks for EAC or ESCC were combined and analyzed for multiplicative effects.

**Results:**

Our analyses includes 327 patients with EAC and 106 patients with ESCC. Low or intermediate activity enzyme genotypes for *GSTM1*, *GSTA1*, *GSTP1 I105V* and *A114V* as well as for *GSTT1*, did not significantly modify the risk for ESCC or EAC in our Dutch population.

**Conclusion:**

Functional genotypes in *GST* genes are not involved in EAC or ESCC susceptibility in Caucasians, in contrast to results on ESCC from Asia or Africa.

## Background

Esophageal Cancer (EC) has limited treatment options resulting in poor 5-year survival rates of 15% [1] and it holds one of the highest cancer mortality rates [2]. The leading global histological subtype used to be esophageal squamous cell carcinoma (ESCC), but in Western countries a rapid increase in adenocarcinoma has occurred over the past decennia [3]. To a large extent this development is due to the increase in prevalence of gastroesophageal reflux disease (GERD) and obesity, two significant determinants of esophageal adenocarcinoma (EAC) [3]. Risk factors as smoking and alcohol consumption are significantly related to ESCC and contribute to the still dominating position of this subtype in Asia [3]. Exposure to environmental risk factors alone cannot explain all cases of esophageal carcinoma, genetic predisposition appears to play a role as well. Detoxification enzymes biotransform carcinogens and toxic agents into less active and water-soluble compounds ready for excretion with bile, urine or faeces. The activity of phase I and II enzymes determine the rate of detoxification of carcinogens in cells and tissues.

Glutathione S-transferases (GSTs) are important phase II biotransformation enzymes catalyzing the nucleophilic addition of glutathione to xenobiotics, oxidative stress products and phase I electrophilic and carcinogenic metabolites [4]. Allelic variation in *GST* genes affects *in vivo* enzyme activity and subsequently decrease the rate of carcinogen detoxification. The GST family has a widely variable organ distribution and four classes of GSTs; Alpha, Mu, Theta and Pi, are known to have esophageal expression [5]. The high level of esophageal expression, polymorphic nature of the *GST* genes and the wide range of carcinogenic substrates, make it all very plausible that this superfamily of detoxification enzymes may influence esophageal carcinoma susceptibility.

Many studies addressing this issue originate from Asia and as a result ESCC has been widely studied as summarized in a recent meta-analysis [6]. To a lesser extend EAC cases have been included in studies, along with Barrett’s esophagus (BE). BE is suggested to be the histological precursor of EAC, displaying metaplastic columnar epithelium and sharing GERD and obesity as risk factors [7]. It was demonstrated that there is a decreased GST enzyme activity or expression following the sequence; normal esophageal epithelium, Barrett’s metaplasia, dysplasia, adenocarcinoma [8,9], suggesting an early etiological role for this enzyme system. Studies examining the role of functional polymorphisms in the *GST* genes expressed in the esophagus (classes Alpha, Mu, Theta and Pi) in relation to EAC risk are lacking. This study was set out to examine whether (combined) *GST* genotypes with altered predicted enzyme activities, modified EAC and ESCC risk in Caucasians. We conducted a case–control study between 2002 and 2012 on 440 patients with EC and 592 age and sex matched controls of the Caucasian race.

## Methods

### Patients with EC and controls

The study was approved by the Medical Ethical Review Committee, region Arnhem-Nijmegen (CMO 2002/114). From 2002 to March 2012, 349 EDTA blood samples from Caucasian patients with esophageal carcinoma were collected from 4 hospitals within 30 km distance in the South-East area of the Netherlands [10]. For some EC cases (n = 91) no blood was available as source of DNA and in these cases DNA was isolated from normal esophageal or gastric tissue, obtained after surgery [10], so in total 440 EC cases could be evaluated. Patients were included in the order of entry to the hospitals. Also 592 EDTA blood samples from healthy controls, matched as a group for age, Caucasian race, gender and geographical location, were recruited after advertisement in local papers, as described earlier [10]. Only patients with a diagnosis of esophageal carcinoma, as confirmed by a pathologist, were included in the study. Tissue, blood and DNA were stored, and DNA isolation was performed as previously described [11].

### Esophageal GST classes and genotyping

GST classes were selected on the basis of possible relevance to esophageal carcinoma etiology, expression in esophageal mucosa, and significance as revealed in Asian case–control studies on ESCC risk [6]. Only functional polymorphisms were chosen for genotyping, again based on possible relevance for EC susceptibility.

Genotypes as well as the sequences of the primers and probes are depicted in Table 1. The GSTM1 isozyme contains three main genotypes at its locus on chromosome 1p13: GSTM1*a, GSTM1*b and GSTM1*0. GSTM1*a and GSTM11*b differ only at base position 519 by a G > C replacement which results in a K > N substitution at codon 173. This has no effect on the catalytic properties of the respective enzymes. However, presence of the GSTM1*0 allele results in a protein absence and homozygosity of GSTM1*0 (*GSTM1* null genotype) results in an absence of enzyme activity [4]. The deletion polymorphism was examined through melt curve analysis [12].

**Table 1:** **Primers and probes and annealing temperatures for detection of *GST *polymorphisms**

**GST**	**Polymorphism *protein change***	***Genotypes***	**Primers (forward) *probes (wild type)***	**Primer (reverse) *probes (mutant)***	**Annealing temp. *MgCl***_***2 ***_***Conc.***
*GSTM1*	Deletion	Wild Type: *GSTM1 +*	5’-CTC CTG ATT ATG ACA GAA GCC-3’	5’-CTG GAT TGT AGC AGA TCA TGC-3’	58°C
	Polymorphism	Hetero.: *GSTM1 +*			*2.0 mM*
		Homoz.: *GSTM1 -*			
*GSTA1*	-631 T > G	Wild Type: GSTA1 *a/*a	5’-TGT TGA TTG TTT GCC TGA AAT T-3’	5’-GTT AAA CGC TGT CAC CGT CC-3’	58.5°C
	-567 T > G	Hetero.: GSTA1 *a/*b			*2.0 mM*
	-69 C > T, -52 G > A	Homoz.: GSTA1 *b/*b			
*GSTP1*	313 A > G	Wild Type*: I-105-I*	5’-CCT GGT GGA CAT GGT GAA TGA-3’	5’-AGC CAC CTG AGG GGT AAG-3’	64°C
	*105 Ile > Val*	Hetero.: *I-105-V*	***5****’-****Fam****-****CGC TGC AAA TAC ATC TCC CTC ATC TAC A****-****BHQ1****-****3****’*	***5****’-****Hex****-****CGC TGC AAA TAC GTC TCC CTC ATC TAC A****-****BHQ1****-****3****’*	*2.5 mM*
		Homoz.: *V-105-V*			
*GSTP1*	341 C > T	Wild Type: *A-114-A*	5’- TGGACAGGCAGAATGGAATAGAG -3’	5’- GGGTCTCAAAAGGCTTCAGTTG -3’	65°C
	*114 Ala > Val*	Hetero.: *A-114-V*	***5****’-****Fam****-****CATCCTTGCCCGCCTCCTGCCAGA****-****BHQ1****-****3****’*	***5****’-****Hex****-****CATCCTTGCCCACCTCCTGCCAGA****-****BHQ1****-****3****’*	*3.0 mM*
		Homoz.: *V-114-V*			
*GSTT1*	Deletion	Wild Type: *GSTT1 +*	5’-TTC CTT ACT GGT CCT CAC ATC TC-3’	5’-TCA CCG GAT CAT GGC CAG CA-3’	59.5°C
	Polymorphism	Hetero.: *GSTT1 +*			*2.0 mM*
		Homoz.: *GSTT1 -*			

The glutathione S-transferase Alpha gene has four functional polymorphisms in full linkage disequilibrium; -631T, -567T, -69C, -52G designated as allele GSTA1*a and -631G, -567G, -69T, -52A designated as allele GSTA1*b. Genotypes were determined via the PCR and restriction enzymes methodology as described by Coles et al*.*[13].

Chromosome 11q13 is the locus for the *GSTP1* gene. A base pair (A > G) substitution at nucleotide 313 results in the amino-acid substitution Isoleucine (I) to Valine (V) at codon 105. In addition, the C > T substitution at nucleotide 341 creates a change in amino-acid transcription at codon 114: Alanine (A) to Valine (V). The *GSTP1* variant alleles express a protein with an altered enzyme activity and substrate specificity [14]. *GSTP1* polymorphisms were determined by using the CFX96 Real Time PCR Detection System (Bio-Rad Hercules, CA, USA). DNA samples were amplified by PCR and detection was performed using sequence specific DNA-probes. Primers and probes for detection of the polymorphisms (Table 1) were designed using Beacon designer software (PREMIER Biosoft International, Palo Alto, CA, USA) and synthesized by Sigma Aldrich (St. Louis, MO, USA). For Real Time PCR, the DNA was denatured at 95°C for 3 min, followed by 40 cycles of 30 sec at 95°C, 30 sec at annealing temperature (Table 1) and 30 sec elongation at 72°C. Analysis was performed by the Bio-Rad CFX Manager detection software for Windows version 2.0 (Bio-Rad Hercules). The intensity of the mutant probe signal (HEX) was plotted against the wild type probe signal.

The *GSTT1* gene is located on chromosome 22q11 and contains 2 variant alleles. The GSTT1*0 allele results in a gene deletion and expresses no enzyme activity, while the wild type allele GSTT1*1 is fully active [15]. The deletion polymorphism was determined via melt curve analysis [15]. However, due to the nature of the polymorphism in *GSTM1* and *GSTT1*, the distinction between heterozygous and homozygous functional genotypes cannot be made by our analyses. Only the homozygous variant genotype (null genotype) can be differentiated.

### Statistical analyses

The independent samples t-test was applied for the differences in continues variables between characteristics of patients and controls. The chi-square test was used for analyzing nominal variables of patient characteristics and to test for differences of frequencies in genotypes between two groups. Logistic regression analyses were used to calculate odds ratios (OR) with 95% confidence intervals (95%CI). Genotypes were classified in predicted enzyme activity groups, and the predicted high enzyme activity group was set as reference. Stratified analyses were performed according to tumor histology.

**Figure 1:**
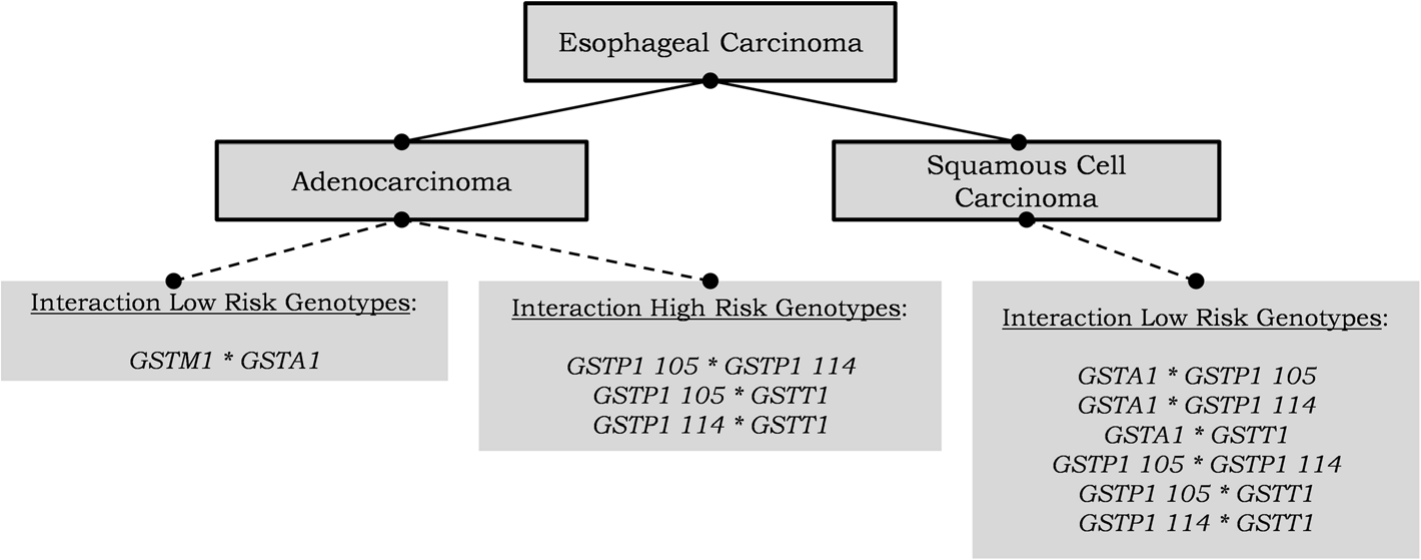
***GST *genotype combinations.** Low risk genotypes: variant genotypes associated with a decreased EAC or ESCC risk (OR < 1.0). High risk genotypes: variant genotypes associated with an increased EAC or ESCC risk (OR > 1.0).

To test the EC risk interactions of GST genotypes, at first the genotypes with predicted low and intermediate enzyme activity of *GSTA1*, *GSTP1 105* or *GSTP1 114* were combined to create two instead of three subgroups: a reference subgroup with predicted high enzyme activity genotypes and a subgroup with predicted low/intermediate enzyme activity genotypes. Then, *GST* genotypes associated with either a decreased risk (OR < 1) or increased risk (OR > 1) were analyzed for interactions (Figure 1) by logistic regression analyses.

All P values were two-sided and a probability level of P < 0.05 was considered to be significant. All analyses were performed with the software SPSS for Windows, version 20.0 (SPSS Inc., Chicago, IL, USA).

## Results

Table 2 shows the demographics of patients and controls. A total of 440 patients with esophageal cancer, 327 with EAC and 106 with ESCC, and 592 healthy controls were included. For 7 patients the exact tumour type was not mentioned in the pathology reports and these samples were therefore excluded from the histology stratified analyses. Controls were matched with the whole EC patient group for Caucasian ethnicity, recruiting area, age and sex. The female sex was more present in patients with ESCC in comparison to patients with EAC (P < 0.001), and in comparison to controls (P = 0.003). Otherwise, no significant difference was found between the reported demographics of patients with EAC or ESSC, and controls.

**Table 2:** Characteristics of patients and controls

**Characteristics**	**Patients**			**Controls**
	**ESCC**	**EAC**	**EC**	
**Number** (%)	106* (24.1%)	327* (74.3%)	440* (100%)	592
**Age** (yrs; mean ± SD)	64.4 ± 10.5	64.9 ± 11.2	64.8 ± 11.1	63.4 ± 11.9
**Gender**				
Male	67* (63.2%)	281 (85.9%)	348 (79.0%)	478 (80.7%)
Female	38* (35.8%)	46 (14.1%)	84 (19.1%)	114 (19.3%)

*Note that for 7 patients data on the exact tumor type are missing, whereas for 1 patient the gender is unknown.

*ESSC* esophageal squamous cell carcinoma, *EAC* esophageal adenocarcinoma.

Genotypes were analyzed for both the histological subgroups in comparison to controls and classified into predicted enzyme activity groups (Table 3). *GSTA1* and *GSTP1* polymorphisms were distributed according to the Hardy-Weinberg equilibrium (*GSTA1* controls/cases, P = 0.71/P = 0.38; *GSTP1-I105V* controls/cases, P = 0.27/P = 0.79; *GSTP1-A114V* controls/cases, P = 0.75/P = 0.97).

**Table 3:** ***GST *genotype distribution according to predicted enzyme activity**

***GST***	***GST *genotypes**	**Predicted enzyme activity**	**ESCC ( *n * = 106)#**	**EAC ( *n * = 327)#**	**Controls *(n* = 592)#**
***GST M1***	*1/*1 & *1/*0	High-Intermediate	48 (45.7%)	156 (47.7%)	273 (46.2%)
	*0/*0	Low	57 (54.3%)	171 (52.3%)	318 (53.8%)
***GST A1***	*1a/*1a	High	43 (41.3%)	131 (42.1%)	214 (37.2%)
	*1a/*1b	Intermediate	50 (48.1%)	133 (42.8%)	277 (48.2%)
	*1b/*1b	Low	11 (10.6%)	47 (15.1%)	84 (14.6%)
***GST P1****I105V*	*Ile / Ile*	High	48 (45.7%)	119 (37.5%)	246 (41.6%)
	*Ile / Val*	Intermediate	42 (40.0%)	157 (49.5%)	261 (44.2%)
	*Val / Val*	Low	15 (14.3%)	41 (12.9%)	84 (14.2%)
***GST P1****A114V*	*Ala / Ala*	High	92 (86.8%)	262 (82.6%)	485 (83.5%)
	*Ala / Val*	Intermediate	14 (13.2%)	52 (16.4%)	91 (15.7%)
	*Val / Val*	Low	0	3 (0.9%)	5 (0.9%)
***GST T1***	*1/*1 & *1/*0	High-Intermediate	87 (82.9%)	248 (75.8%)	463 (78.3%)
	*0/*0	Low	18 (17.1%)	79 (24.2%)	128 (21.7%)

*ESSC* esophageal squamous cell carcinoma, *EAC* esophageal adenocarcinoma.

#Some genotyping data were missing due to PCR bias.

Table 4 depicts the odds ratios for the comparisons of groups with predicted enzyme activity between patients with ESCC or EAC, and controls. For *GSTM1*, *GSTA1*, *GSTP1 I105V* and *A114V* as well as for *GSTT1*, the low or intermediate activity enzyme genotypes did not significantly modify the risk for ESCC or EAC in our population.

**Table 4:** Odds ratios and 95% CI according to predicted GST enzyme activity genotypes for ESCC and EAC patients compared to controls

***GST***	**Comparisons**	ESCC OR (95%CI)*	**EACOR (95%CI)**
***GST M1***	High- Intermediate	Ref	Ref
	Low	1.06 (0.69 – 1.61)	0.94 (0.72 – 1.23)
***GST A1***	High	Ref	Ref
	Intermediate	0.88 (0.56 – 1.38)	0.78 (0.58 – 1.06)
	Low	0.58 (0.28 – 1.21)	0.91 (0.60 – 1.39)
***GST P1*** *I105V*	High	Ref	Ref
	Intermediate	0.83 (0.53 – 1.29)	1.24 (0.93 – 1.67)
	Low	0.92 (0.49 – 1.72)	1.01 (0.66 – (1.56)
***GST P1*** *A114V*	High	Ref	Ref
	Intermediate	0.86 (0.47 – 1.59)	1.06 (0.73 – 1.54)
	Low		1.11 (0.26 – 4.68)
***GST T1***	High-Intermediate	Ref	Ref
	Low	0.80 (0.46 – 1.39)	1.15 (0.84 – 1.59)

* Odds ratios were adjusted for gender.

*ESSC* esophageal squamous cell carcinoma, *EAC* esophageal adenocarcinoma.

**Figure 2:**
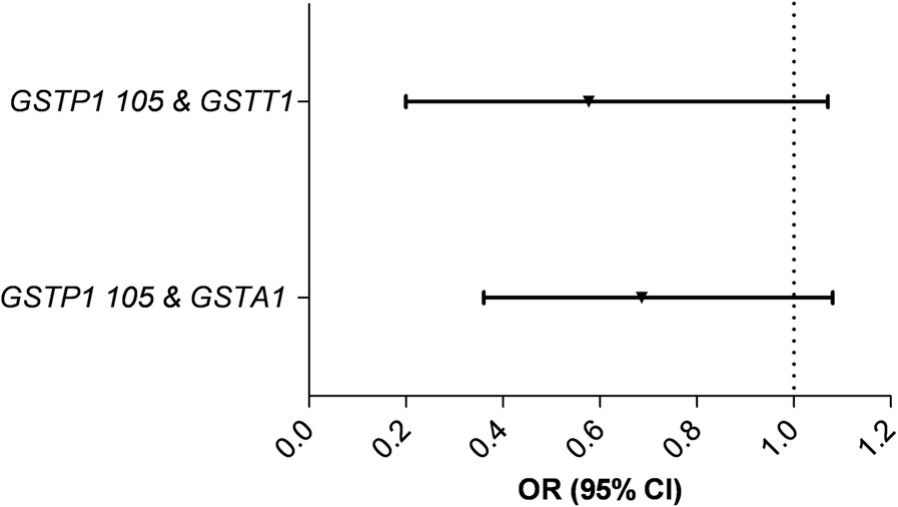
**Combined low risk genotypes and ESCC susceptibility.** ESCC = Esophageal Squamous Cell Carcinoma; OR = Odds Ratio; 95%CI = 95% Confidence Interval. *GSTP1 105* = low and intermediate activity *GSTP1 105* genotypes; *GSTA1* = low and intermediate activity *GSTA1* genotypes; *GSTT1* = low activity *GSTT1* genotype.

The (combined) genotypes of predicted low and low/intermediate enzyme activity (see Table 3) were analyzed for interactions, when genotypes had similar effect sizes (decreased or increased risk). For example, the correlation between the combined genotypes of *GSTM1* &*GSTA1* was analyzed because the predicted low enzyme activity genotypes of *GSTM1* or low/intermediate genotypes of *GSTA1* both showed an OR < 1 for EAC risk (see Table 4 and Figure 1). Only the correlation of predicted low/intermediate enzyme activity genotypes *GSTP1 105* and *GSTA1* (*P* < 0.05) and *GSTP1 105* and *GSTT1* (*P* = 0.053) showed a significant and near significant lower risk for ESCC, respectively. The genotypes of predicted low and intermediate *GSTP1 105* & low and intermediate *GSTA1* enzyme activity, and of low and intermediate *GSTP1 105* & low *GSTT1* were combined and set off against their corresponding predicted high activity genotypes, but the associations for ESCC risk failed to reach significance; OR 0.62; 95%CI 0.36 – 1.08 and 0.46; 0.20 – 1.07, respectively (Figure 2).

## Discussion

This case–control study reveals no associations between modified activity *GST* genotypes and EAC or ESCC susceptibility in Caucasians. Only *GSTA1* low or intermediate genoytpe was associated with a non-significant protective effect for both tumour types, whereas the remaining GST classes showed contradictory effect sizes for EAC and ESCC.

Although many case–control studies investigated *GST* polymorphisms and EC risk, only seven originated from Western populations and reported on EAC risk. These studies were from Europe [16-19], the USA [20] and Canada [21,22]. Our results contrasts with the findings of Casson et al. [21] and Abbas et al. [16] that the *GSTP1 Ile105Val* and GSTT1*1*1 genotype increased EAC risk, ORs are 2.5; 1.0 – 6.3 and 13.3; 1.7 – 106.9, respectively. Furthermore van Lieshout *et al*. [18] and Zendehdel *et al*. [19] reported on both ESCC and EAC susceptibility and found the *GSPP1* variant allele to increase the risk for EAC and ESCC, respectively. However, all studies offer inconclusive results due to very low number of EAC and ESCC cases (*n* < 100), except for the study of Murphy et al. which included 207 EAC cases [17]. Our study includes a relatively large EAC group and consequently may offer a higher power.

Although there is a degree of inconsistency and population sizes are relatively small (n = 45 and n = 234 [23,24]), ESCC risk is generally addressed by Asian studies and reported increasing ESCC risk for the homozygous *GSTP1105Val* and homozygous GSTM1*0 genotypes whereas some studies even conclude them to be independent risk factors for ESCC in China [25-27] and Brazil [28]. The largest studies however (n = 562 & n = 245; [29,30]), originated from South Africa with conflicting results, as mixed ancestry in African populations are a difficulty for genetic investigators. So although results differ globally, our finding is in accordance with several meta-analyses [6,31-33], concluding that *GST* genotypes do not seem EC risk factors, the variant *GSTP1 105* genotypes excluded [33]. Zendehdel *et al*. conducted a Swedish case–control study as well as a meta-analysis, including only studies with Caucasians, and stratified according to histology [19]. Interestingly, this meta-analysis consisted of the Caucasian studies mentioned above [16-18,20-22] and found that the *GSTP1 I105V* polymorphism was associated with an increased risk for ESCC (1.4; 1.0 – 2.2) and not for EAC (1.2; 0.9 – 1.6). However, their own data contributed largely to this effect, as their patient numbers consisted 60% of the pooled numbers of the meta analysis.

So according to the literature so far, only the *GSTP1 Ile105Val* polymorphism seems involved in ESCC etiology. Our genotype-genotype interaction analyses confirmed this premise, as combinations of low and intermediate *GSTA1*, or low *GSTT1*, with low and intermediate *GSTP1 105* genotypes showed a trend for a decreased ESCC risk. Other studies on interactions mostly examined the *GSTM1* null genotype in combination with either *CYP2E1*[25,26,34] or *CYP1A1* genotypes [27]. Moaven et al. reported that interactions between *GST* polymorphisms were not associated with a modifying effect for ESCC in an Iranian population [35], while Wang *et al*. reached similar conclusions in a Chinese cohort [36]. Both studies however had small patient numbers (*n* = 148 and *n* = 107, respectively). Larger study sizes are warranted to investigate the effect of combined *GST* genotypes, more explicitly combinations with *GSTP1* genotypes, and the risk for ESCC. Moreover, GSTP1 is the main GST enzyme expressed in the esophagus [9] and the genetic variants express proteins with a large reduction of enzyme activity [37].

Another important finding clearly depicted by this study, is the contrasting effects for ESCC and EAC risk, possibly related to their different etiologies. An increasing risk for EAC by *GST* genotypes may be due to a lower detoxification rate of carcinogens. But studies originating from South Africa [30] and Iran [38] correspond with our results and also report protective effect sizes of *GST* variants for ESCC risk. Matejic et al. explains this by the prevention of glutathione (GSH) depletion, due to a decreased conjugation activity of the GSTs. GSH is a powerful antioxidant and thus an optimal GSH level to protect against oxidants is maintained [30]. However, it remains difficult to clarify the difference in effect size between EAC and ESCC. Substrate overlap between GST isozymes can compensate the decreased detoxification of mutagens attributable to a single enzyme deficiency. Moreover, the risk for EAC and ESCC is probably substrate dependent. Although substrate overlapping occurs for oncogenetic compounds such as Benzo A-Pyrene-Diol-Epoxide, 1,2-epoxy-3-butene and 1,2:3,4-diepoxybutane, there is a degree of specificity per GST isoenzyme. The Mu, Alpha and Pi GSTs conjugate several carcinogenic epoxides like aflatoxin B1 epoxide (a naturel carcinogen) and polycyclic aromatic hydrocarbons diol epoxide such as benzo[a]pyrene (BaP). The Theta class GST is involved in the detoxification of small dihaloalkanes such as dichloromethane which is an important compound used in paint strippers, plastics and pharmaceutical drugs, and also dibromoethane, an anti-knocking agent in gasoline.

Limitation of our study are the relatively small number of cases involved, which after stratification according to tumor histology, results in even lower subgroup numbers and therefore limits the power of the study. For instance, interactions with the *GSTP1* variants showed a tendency to modify ESCC risk, but genotype combinations failed to reach statistical significance, which mainly may be a power issue as the especially the ESCC group is small in our Dutch Caucasian population. Another limitation is the lack of data on exposure to mutagenic compounds (smoking, alcohol consumption, etc.) in patients and controls, to counterbalance confounding effects (and additionally examine potential gene-environment effects).

## Conclusion

We conducted the largest case–control study so far on *GST* variant genotypes and esophageal cancer risk in a Western population of Caucasian ethnicity. Although this study did not detect significant associations between altered predicted enzyme activity *GST* genotypes and EAC or ESCC risk, our results indicate that gene-gene interactions between *GSTP1* variants could play a role in EC susceptibility.

## Competing interests

The authors declare that they have no competing interests.

## Authors’ contributions

PD, JPHD and WHMP designed the study. PD, JS, RHMtM, HMJR, JOK, TW, BJMW, ACITLT and WHMP were involved in acquisition, analysis and interpretation of data. PD and WHMP drafted the manuscript, which was critically revised by JS, RHMtM, HMJR, JOK, TW, BJMW, ACITLT and JPHD. All authors read and approved the final manuscript.

## Pre-publication history

The pre-publication history for this paper can be accessed here:

http://www.biomedcentral.com/1471-230X/13/97/prepub
